# Supplementary material for: An examination of school reopening strategies during the SARS-CoV-2 pandemic
Source: PLoS One. 2021 May 20;16(5):e0251242. doi: 10.1371/journal.pone.0251242 (PMC8136712; doi:10.1371/journal.pone.0251242)
Supplement: S1 File — (DOCX) [file pone.0251242.s002.docx]

**S1 File**

**Compartmental model.** We let $S\left( t \right)$, $E\left( t \right)$, $I\left( t \right)$, and $R\left( t \right)$ denote the fraction of susceptible, exposed, infected, and removed individuals, respectively, in the overall population at time $t$. Infecteds may or may not present with symptoms. The removed pool accounts for individuals with negligible contribution to infection spread, including individuals that have either recovered with full immunity or are dead. For the time periods we examine, we make the plausible assumption that individuals do not return to the susceptible pool once infected.^1^

Denote by $I_{il}\left( t \right)$ the fraction of infected in age group $i$ and cohort $l$. We propose the following model for the force of infection on individuals in age group $j$ and cohort $k$:

$$\text{force of infection}= \left( \text{interaction between cohorts }k\text{,}l \right)\times\left( \text{transmission between age groups }i\to j \right)\times\left( \text{fraction of infecteds in age group }i,\text{ and cohort }l \right)$$

$$\lambda_{jk}\left( t \right)=\sum_{\mathcal{l}} \sum_{i} \alpha_{kl}\times\beta_{ij}\left( t \right)\times I_{i\mathcal{l}}(t)$$

For pairs of cohorts $k\mathcal{\neq l}$, the choice $\alpha_{k\mathcal{l}}=0$ reflects complete separation, whereas $\alpha_{k\mathcal{l}}=1$ corresponds to complete mixing without mitigating policies. Values within these limits may be interpreted as decreased interaction due to physical or social distancing. We allow weak cohort interaction ($\alpha_{k\mathcal{l}}=0.05$, $k\mathcal{\neq l}$) in all our simulations. To capture the variability in exposure to infectious individuals across age groups and different cohorts, the transmission rate $\beta_{ij}\left( t \right)$ is time-inhomogeneous. These choices effectively model the density dependence of coronavirus transmission on cohort isolation and contact patterns between different age groups. The ordinary differential equations (ODE) describing cohort $k$ is given by

$$\begin{matrix} \frac{dS_{1k}}{dt}= & -\lambda_{1k}S_{1k} & & & \frac{dS_{2k}}{dt}= & -\lambda_{2k}S_{2k} \\ \frac{dE_{1k}}{dt}= & \lambda_{1k}S_{1k}-\sigma_{1}E_{1k} & & & \frac{dE_{2k}}{dt}= & \lambda_{2k}S_{2k}-\sigma_{2}E_{2k} \\ \frac{dI_{1k}}{dt}= & \sigma_{1}E_{1k}-\gamma_{1}I_{1k} & & & \frac{dI_{2k}}{dt}= & \sigma_{2}E_{2k}-\gamma_{2}I_{2k} \\ \frac{dR_{1k}}{dt}= & \gamma_{1}I_{1k} & & & \frac{dR_{2k}}{dt}= & \gamma_{2}I_{2k}, \end{matrix}$$

with the left and right columns corresponding to children and adults, respectively.

**Basic reproductive number.** We characterize the basic reproductive number $R_{0}$ indicative of growth potential of an infectious disease. Specifically, $R_{0}$ quantifies the expected number of secondary infections due to a single infected within a completely susceptible population. The threshold $R_{0}$ value of 1 marks the boundary between explosive growth ($R_{0}>1$) and decline of an epidemic to extinction ($R_{0}<1$). We characterize $R_{0}$ using the next generation method as outlined by Diekmann, Heesterbeek, and Robers.^2^ Under the assumption that viral infections have been sufficiently contained in the community prior to reopening, it is reasonable to linearize dynamics by taking $S\left( 0 \right)\approx1$. Thus, the transmission and transition operators $\boldsymbol{T}$ and $\boldsymbol{\Sigma}$ are given respectively by the matrices

$$\boldsymbol{T}=\left[ \begin{matrix} 0 & \alpha_{11}\beta_{11}S_{11} & 0 & \alpha_{11}\beta_{12}S_{21} \\ 0 & 0 & 0 & 0 \\ 0 & \alpha_{11}\beta_{21}S_{21} & 0 & \alpha_{11}\beta_{22}S_{21} \\ 0 & 0 & 0 & 0 \end{matrix} \right] \boldsymbol{\Sigma}=\left[ \begin{matrix} -\sigma_{1} & 0 & 0 & 0 \\ \sigma_{1} & -\gamma_{1} & 0 & 0 \\ 0 & 0 & -\sigma_{2} & 0 \\ 0 & 0 & \sigma_{2} & -\gamma_{2} \end{matrix} \right],$$

based on the infectious subsystem defined by $\boldsymbol{x}=\left[ E_{1},I_{1},E_{2},I_{2} \right]^{\top}$ for a single cohort. Here the subscripts 1 and 2 denote children and adults, respectively. Together, these linear operators define an embedded subsystem that completely characterizes infectious dynamics, namely $dx/dt=\left( \boldsymbol{T}+\boldsymbol{\Sigma} \right)\boldsymbol{x}$. The standard theory identifies $R_{0}$ as the spectral radius of $-\boldsymbol{T}\boldsymbol{\Sigma}^{-1}$, a quantity computed numerically. In the case of multiple cohorts, the structures of $\boldsymbol{T}$ and $\boldsymbol{\Sigma}$ as given are repeated in a tiled fashion, with the appropriate changes in indices for $\alpha_{k\mathcal{l}}$ and $S_{j\mathcal{l}}$.

**References**

1. Seow J, Graham C, Merrick B, et al. Longitudinal evaluation and decline of antibody responses in SARS-CoV-2 infection. *medRxiv*. Published online July 11, 2020:2020.07.09.20148429. doi:10.1101/2020.07.09.20148429

2. Diekmann O, Heesterbeek JAP, Roberts MG. The construction of next-generation matrices for compartmental epidemic models. *J R Soc Interface*. 2010;7(47):873-885. doi:10.1098/rsif.2009.0386
